# Multifunctional Membranes—A Versatile Approach for Emerging Pollutants Removal

**DOI:** 10.3390/membranes12010067

**Published:** 2022-01-03

**Authors:** Ecaterina Matei, Cristina Ileana Covaliu-Mierla, Anca Andreea Ţurcanu, Maria Râpă, Andra Mihaela Predescu, Cristian Predescu

**Affiliations:** 1 Faculty of Materials Science and Engineering, University Politehnica of Bucharest, 313 Spl. Independetei, 060042 Bucharest, Romania; ecaterinamatei@yahoo.com (E.M.); rapa_m2002@yahoo.com (M.R.); andra.predescu@upb.ro (A.M.P.); cristian.predescu@upb.ro (C.P.); 2 Faculty of Biotechnical Systems Engineering, University Politehnica of Bucharest, 313 Spl. Independentei, 060042 Bucharest, Romania; 3 Center for Research and Eco-Metallurgical Expertise, Faculty of Materials Science and Engineering, University Politehnica of Bucharest, 313 Spl. Independetei, 060042 Bucharest, Romania; anca.turcanu@upb.ro

**Keywords:** membranes, electrospinning, emerging pollutants

## Abstract

This paper presents a comprehensive literature review surveying the most important polymer materials used for electrospinning processes and applied as membranes for the removal of emerging pollutants. Two types of processes integrate these membrane types: separation processes, where electrospun polymers act as a support for thin film composites (TFC), and adsorption as single or coupled processes (photo-catalysis, advanced oxidation, electrochemical), where a functionalization step is essential for the electrospun polymer to improve its properties. Emerging pollutants (EPs) released in the environment can be efficiently removed from water systems using electrospun membranes. The relevant results regarding removal efficiency, adsorption capacity, and the size and porosity of the membranes and fibers used for different EPs are described in detail.

## 1. Introduction

The application of electrospun membranes for wastewater treatment has been the subject of numerous review papers, and their application for the removal of emergent pollutants has been studied in recent years in several review papers, with their numbers constantly increasing [1]. The data regarding nanofibers presented in this paper cover developments from 1998 to 2021. Because the significance of these emerging pollutants is increasing, the aim of this literature review is to contribute to developing a possible database covering their decontamination performance. Thus, this paper presents tested membrane types along with their characteristics, as well as concrete results for different classes of emerging pollutants.

Today, among the many environmental risks, the water crisis is one of the most important. Thus, it is imperative that a technological approach be developed to deliver high-quality standards for water and welfare. Appropriate treatment methodologies should limit the presence of pathogens, toxic chemicals, pharmaceuticals, heavy metals, fertilizers, and endocrine disruptors, as emergent pollutants in water [2,3,4,5,6]. For these types of pollutants, classical water treatment methods are insufficient for achieving efficient degradation, and thus researchers today are focused on hybrid technologies in which classical methods are combined with hybrid techniques such as chlorination and UV radiation [7]. The results are not yet technologically and economically effective, and the challenge of producing high-quality water remains an issue.

One of the most significant issues regarding the presence of emergent pollutants in the environment is their persistence and their risk of diffusing directly from the soil to groundwater, making their treatment more complex. Today there are several membrane-based techniques, such as microfiltration (MF), ultrafiltration (UF), nanofiltration (NF), reverse osmosis (RO), and forward osmosis (FO). Any of these techniques can be integrated into hybrid water treatment systems, but there is currently only a small number of such applications on an industrial scale.

There are numerous data on the experimental parameters by which membranes based on electrospun fibers can be obtained, including the initial concentration and type of polymer and solvent, electrospinning speed, temperature, type of collector, and electrospinning method. This work highlights the performance that these types of materials can have in the removal of some emerging pollutants, for which viable solutions are still being sought with respect to decontamination, and also in the integration of solutions found in existing decontamination systems. Thus far, as will be shown in the course of the paper, the basic purpose, at the application level, is to integrate these materials as a support for thin film composites (TFC) that subsequently form membranes used in separation processes. In recent years, these types of materials have been experimentally tested in single adsorption or hybrid processes in which they have been combined with advanced oxidation or photocatalytic techniques.

This review article outlines the performances of electrospun membranes when applied for the removal of emergent pollutants from waters, based on their nanofiber properties and efficiency. This paper also includes a comprehensive discussion of membrane materials as advanced materials with high characteristics associated with filtration processes dedicated to emergent pollutants.

## 2. Emerging Pollutants

### 2.1. Classification

Water pollution still represents an urgent issue at the global level, with respect to both quality and quantity [2,8]. Emerging pollutants, according to the Norman Substance Database, are defined as synthetic or natural pollutants that should potentially be included in future regulations due to their ecotoxicity and impact on life and the environment, that have not yet been introduced into routine monitoring plans [9,10]. Emerging pollutants can be classified, according to their physico-chemical properties, into the following categories: organics (such as pharmaceuticals, industrial chemicals, pesticides), inorganics (such as trace metals), and contaminating particles (such as nanoparticles and microplastics) [3,8].

There are two different pollution sources from which emerging pollutants can be released into the environment: through wastewater treatment plants in urban or industrial areas (as point sources), and through atmospheric deposition, crops, and animal production (diffuse sources).

According to Stone, when an emerging pollutant is defined as a contaminant, this may either be as a result of the identification of a new source or pathway into the human body, or as a result of the development of a new detection method or treatment process [11].

According to the EPA, those pollutants recently discovered, often as a result of improvements in analytical detection performance, and which are not necessarily new chemicals, often found in the environment but not monitored until recent years, are defined as “contaminants of emerging concern” [12]. Thus, there are more than 20 classes of compounds under the umbrella of emerging pollutants, among which, we can mention:

- Persistent organic pollutants (POPs) from flame retardants, furniture foam, plastics, etc.;
- Pharmaceuticals and personal care products (PPCPs) from prescribed drugs (antidepressants, blood pressure, etc.) to over-the-counter medications (ibuprofen, acetaminophen, etc.), as well as bactericides (such as triclosan), sunscreens, and synthetic musks;
- Veterinary medicines such as antimicrobials, antibiotics, antifungals, and growth hormones;
- Endocrine-disrupting chemicals (EDCs), including synthetic estrogens and androgens, and naturally occurring estrogens, along with organochlorine pesticides and alkylphenols, well-known to alter normal hormonal functions and steroidal synthesis in aquatic organisms;
- Nanomaterials such as carbon nanotubes or nano-scale particulate titanium dioxide, with little being known about either their environmental fate or effects.

All these products contain compounds that have a high probability of being concentrated in biological species and transferred to the food chain. Their identification has raised an urgent need to establish efficient removal technologies that are technically and economically feasible. Their presence in domestic and industrial streams has demonstrated that current conventional water and sludge treatment plants are not able to provide the required efficiency [13].

**Figure 1:**
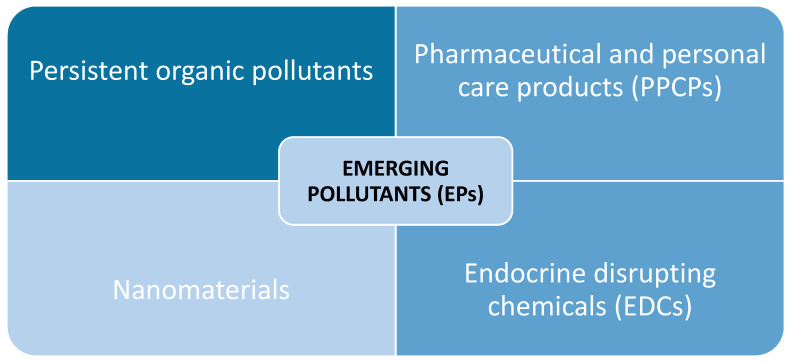
Influence of emerging pollutants (EPs) on environments.

These emerging pollutants represent a constant issue due to the high rate of urbanization, consumption trends, and industrial technologies, all of which are focused on elevated standards of life quality [10]. The main categories of emerging pollutants (EPs) that influence aquatic, air and terrestrial environments are presented in Figure 1. Thus, new products appear on the market as a result of society’s requirements. Along with these, the development of new analytical methods for the correct detection of the substances that are part of these products, and the high costs involved, represent an additional challenge [10,14,15]. On this basis, advanced water treatment methods can be identified and integrated into hybrid systems alongside classical methods. Because membrane systems that involve a filtration step are essential, the materials underlying the design of filters and membranes are also of increasing interest.

There are plentiful data regarding the occurrence, sources, behavior, impacts, and risks of emerging pollutants in water, sediments, soil, and the atmosphere [14,16,17], but with little data from investigations of their toxicity. The most studied emerging pollutants are endocrine disruptors, and the most frequently detected in the environment are pharmaceutical products (CECs), personal care products (PPCPs), and flame retardants. According to the WHO, one of the biggest food safety and health problems is antibiotic resistance, especially where these can be acquired without a prescription, and thus their spread and resistance has become noticeable in the environment [18,19]. Antibiotics can cause some bacteria to become resistant to low concentrations of these classes of substances, so removing them from environments is becoming even more complicated [20].

On the list of emergent pollutants, another category consists of personal care products (PCPs), comprising especially ultraviolet radiation screening compounds or organic UV filters [21]. Their intensive use has caused them to be actively introduced into waters (rivers, lakes, seawater, groundwater, sediments, and biota) through recreational activities. Most of them are detected in wastewater treatment plants, where the actual treatment steps are not adequate to remove them.

### 2.2. Environmental Impact

All these above-mentioned aspects related to the persistence in the environment and the occurrence in water treatment systems of these types of pollutants have been studied in recent years in order to solve the deficiencies occurring in conventional treatment systems. The literature indicates that membrane technologies, activated sludge technologies, sorption processes, advanced oxidation processes, phytoremediation, and bioremediation are processes suitable for the removal of emerging pollutants. Each of these processes has demonstrated advantages and disadvantages, depending on the category of emerging pollutant being removed from the system, the complex matrix, and the level of concentration. The main advantages and future challenges regarding conventional and non-conventional processes applied for EP removal are presented in Table 1.

The efficiency of their removal from wastewater effluent is related to the risk of their appearance in surface water, sediment, soil, groundwater, and seas [20,22,23]. The challenge arises when concentrations are low and these substances are present in complex matrices (micro- or nanograms per liter) [3], so in addition to analytical methods that must be sufficiently sensitive for proper trace detection, studies on ecotoxicology, risk assessment, and spatial distribution must be performed to establish effective treatment methods for their removal from contaminated aqueous systems [20].

Today’s technologies for water/wastewater treatment are adequate and cost effective in specific applications [24]. Often, wastewater contains a variety of compounds, including metals, microorganisms, organic compounds, as natural or synthetics (pharmaceuticals), and a single technology is unable to meet the required quality standards associated with a circular model of water management [25]. When there are effective methods (such as chlorination for drinking water, reverse osmosis for the desalination of seawater, and activated sludge for organic matter, phosphorus, and nitrogen), in the case of a complex water matrix, treatment plants may employ various techniques in combination to remove heavy metals, solid suspensions, and persistent organic pollutants. Thus, hybrid technologies can provide the efficiency and quality required by water standards [26]. Thus, hybrid systems combining active sludge, as a classical method, with membrane systems based on ultra-, micro-, or nanofiltration offer effluent quality, containing solid suspensions, organics, and nutrients [20]. Several hybrid configurations adapted to emerging pollutants have been tested at pilot level in the EU, and represent a new generation of water technologies, but these are associated with considerable investment costs, delaying broad application [27,28]. In order for these systems to meet the requirements regarding efficiency, cost, and environmental impact, the experimental conditions must coincide with real ones in terms of parameters, whereas to date, the literature includes some notable differences. Additionally, given the performance of membrane systems, these need to be further developed. The types of materials underlying these membranes are important in their manufacture, being responsible for the efficient removal of pollutants. Importantly, in the case of the degradation of these emerging pollutants, the compounds resulting from the degradation can sometimes be more hazardous than the original ones, leading to high ecotoxicity, as water treatment systems are not always efficient. This phenomenon occurs especially in the case of advanced oxidation systems, and such cases have been described in the literature [20,29,30,31,32].

## 3. Membranes

### 3.1. Treatment Technologies for Electrospun Functional Membranes

The technologies applied for the preparation of electrospun membranes are designed to introduce new functions to the membrane, such as excellent hydrophobicity and mechanical properties, as well as chemical stability, in order for the membrane to be used in the treatment of emerging pollutants in water [33].

Electrospun functional membranes are the subject of pretreatment and post-treatment treatment technologies, after the preparation step. By means of pretreatment tehcnologies, other functional components can be added directly to spinnable polymer solutions in order to provide membrane functionality. The solvent and functional components have to be compatible, and the dispersion of functional solid components in the working polymer solution should also be considered [34]. Conversely, precipitation and possible clogging of the spinneret could take place, and thus, viscosity, solubility, and dispersion are compulsory parameters to control [35,36].

Post-treatment technologies involve the treatment of the membrane (calcination treatment or surface coating) to obtain a large specific surface area, while hydrophilic functional groups on the surface of the fibers extends the possibilities of performing water treatment using certain hydrophobic polymers that possess good mechanical strength [34,37]. The post-treatment could be a physical modification, but without a total change in fiber properties. Additionally, chemical modification is applied to enhance the internal fiber properties; for example, the functionalization of cellulose nanofibers using sulfhydryl group can be successfully performed by means of the deacetylation of electrospun cellulose acetate nanofiber membrane, followed by temperature treatment (at 80 °C for 22 h) and the esterification of hydroxyl groups with 3,3′-dithiodipropionic acid and the additional reductive cleavage treatment of the disulfide bond [38].

The pretreatment and post-treatment technologies can be combined; for example, an easily recycled photocatalyst for antibiotics degradation can be synthesized using dispersed graphitic carbon nitride (g-C_3_N_4_) embedded in polyethylene terephthalate (PET) by electrospinning with subsequent hydrothermal treatment [39].

The role of a membrane is indicated by its selectivity with respect to pollutant separations resulting from their transport between two phases [40,41]. Depending on the pore structures of materials, membranes can be classified as porous or dense (non-porous) [42].

### 3.2. Dense (Non-Porous) Membranes

Focusing on environmental applications, membrane biofilm reactors are designed within non-porous-based materials [43,44]. Flat-sheet membranes with pore sizes lower than 0.2 nm are considered to be non-porous membranes [43]. Today, composite non-porous membranes from the polyurethane layer between polyethylene micro-porous layers are commercially available, because porous layers usually offer structural support to thin non-porous membranes [44]. One of the most significant features of these membranes is their ability to demonstrate gas transfer resistance only if a thin layer is present. Some applications include the removal of organic compounds from contaminated gas streams, with good results being obtained in benzene removal using latex non-porous membrane [45]. Usually, dense-phase materials (for example, silicone rubber) are used for the design of semipermeable hydrophobic membranes.

For water decontamination, in order to separate impurities, oils, organics and biological species, and other emerging pollutants, purification methods are classified, on the basis of the pore size of the materials used, into nano- and micro-porous methods [46]. Nano-porous membranes act as a dense film with a thickness of only a few hundred nanometers, in which pollutant molecules are separated through a solution–diffusion process directed by pressure, concentration, and potential gradient across the membrane [40,47]. These types of membranes are applied in processes in which reverse osmosis (RO), forward osmosis (FO), nanofiltration (NF), and membrane distillation (MD) are used [48].

NF and RO membranes are applied in desalination water treatments, where high pressure is necessary compared with FO [40,49]. A very crucial aspect when polymeric membranes are used, especially for NF, is the accurate control of their morphology, as well as their chemical, thermal and mechanical integrity, in order to completely remove target pollutants without affecting the permeate flux [50,51]. Under these considerations, thin-film composite (TFC) membranes have been used extensively in NF applications. These TFC membranes are designed on an asymmetric porous support obtained using the phase inversion method [52]. Recently, conventional support systems in TFC membranes have been replaced with electrospun scaffolds that exhibit excellent interactions between the barrier and support layers, improving the separation efficiency [40,51,53,54,55,56,57]. Electrospun structures offer thin layers with good mechanical properties, and could also be a reliable option for FO and, combined with electrospun scaffolds, could lead to high flux due to the interconnected pore structure [33,58,59,60,61,62].

### 3.3. Porous Membranes

Porous membranes are usually used for micro (MF) and ultrafiltration (UF) applications, where the main characteristic is the pressure acting on the membrane, and only the smallest particles pass through it. In the case of UF, the membrane is able to retain particles greater than 0.001 μm in size [63]. As in the case of dense nano-membranes, the pore size and the distribution are crucial features necessary for mechanical strength.

Sundarrajan and colleagues indicated that conventional UF membranes can be integrated into TFC-type configurations, which incorporate three layers: (i) a micro-fibrous nonwoven support for mechanical strength, (ii) a UF membrane for assuring the permeate flow resistance, coated with (iii) a thin film as a barrier layer for solute exclusion and flux rate monitoring [46].

Another application of micro-porous membranes is in membrane distillation (MD) processes, where the water is maintained on one side, and the vapors cross through the membrane pores. This MD process requires hydrophobic membranes that possess a narrow pore size distribution, good mechanical strength, and high values for liquid entry pressure (LEP) [64,65,66]. The phase inversion technique is one of the most facile techniques used as a preparation method, and possesses the advantage of obtaining a large variety of pore sizes in accordance with polymer type and concentration, as well as precipitation method and temperature [43]. Electrospun membranes are appropriate candidates for MD processes, due to their effectiveness in the control of material characteristics and design [40,64].

## 4. Electrospinning Technique

### 4.1. Principles, Characteristics, Parameters

Electrospinning is a technique that has been known since ancient times, and electrospun shapes exist even in nature [67]. Xue et al. mention in their review the history of this technique’s emergence, with the most common examples in nature being the spider’s feathers, which have diameters of between 2 and 5 μm, or the filaments of cocoons built by silkworms [68]. Nature has thus been a source of inspiration for man, with the textile industry’s achievements in wool and cotton yarn being one example. Progress in chemistry, particularly in the polymer industry, has led to the development of synthetic fibers, with nylon representing the greatest advance in this field. Subsequently, numerous synthetic fibers have been developed using wet, dry, melt, or gel methods [69,70].

Today, electrospun nanofibers have applications in areas such as the textile industry [71,72,73,74], medicine [75,76,77,78,79], sensor manufacturing [80,81], cosmetics [82,83,84], and water purification [40]. In short, obtaining fibers using the wet method involves placing an extruded polymer solution from a spinneret into a chemical bath, where it is solidified into fibers as a result of a chemical reaction or dilution. In the dry method, the fibers are formed as a result of the extrusion of the polymer through the air onto a surface, with the solvent of the solution being evaporated into the atmosphere by gentle heating or airflow. In the case of the melt method, the fibers are formed by cooling, with the generation of extruded polymer from the spinneret. To obtain fibers with special properties, mainly high mechanical strength, a polymer solution gel is used, which is able to form fiber by drying in air and then in a liquid bath. Depending on the application, these types of methods can be chosen to obtain submicron-sized, high-strength fibers.

The arrangement of the fibers obtained by electrospinning results in the formation of membranes, the main advantage of which is the small diameter of the fibers, but also the low pore size, which induces a large specific surface area, high porosity, surface roughness, and low weight [34,68]. The advantages of such a membrane also arise from the ease with which it can be functionalized/decorated later with different compounds with advanced properties for use in fields in which these types of membranes are applied. Thus, nanofiber-based composites may emerge that are more efficient than bare nanofibers (e.g., physical, chemical, and catalytic properties can be enhanced).

The principle of the electrospinning technique is really simple. A high-voltage device, a spinneret, and a collector are required for fiber formation [68,85]. Polymer droplets upset the surface tension, and as they exit the spinneret, they form one or more ultrafine jets that are captured on a collector device as thin fibers.

Applications have proven the need to obtain fibers with controlled morphology; as such, the parameters are driven by system, processing, and environmental factors [86]. Fibrous structures display important characteristics useful for many applications, especially when nanoscale diameters are obtained. Thus, fabrication techniques remain a challenge, with a focus on nanofibers. Nowadays, the electrospinning technique is designated as an emerging technique that offers good control and operating conditions for the production of highly porous smooth non-woven structures.

In contrast to traditional methods of manufacturing phase inversion membranes, those obtained by electrospinning show a structure with a relatively uniform pore size distribution, with high interconnectivity between the pores and with a high porosity of about 80% [40,87,88,89]. These arguments support the use of such membranes in separation processes with fiber diameter sizes in the nanometer range.

Nanofibers represent a nexus class of nanomaterials with exceptional properties due to their nanometric scale and high specific surface activity.

Electrospun membranes are effective in water purification processes, including membrane distillation (MD) and pretreatment steps prior to reverse osmosis (RO) or nanofiltration (NF), with the effect of removing divalent metal ions, oils, and other contaminants. Thin film composite (TFC) membranes for reverse osmosis (RO) and nanofiltration (NF) desalination are often fabricated on electrospun polymer supports [46,90].

There are numerous data on the practical applicability of such membranes in air and water filtration [91,92,93], with focus on membrane architecture, new types of materials, synthesis and characterization methods for simple or functionalized membranes, especially in case of membrane distillation (MD) processes [68], and or other water treatment processes [94]. Post-treatment methods have also been intensively studied [95]. Developments in recent years in the production of electrospun membranes have been described in detail, especially for membrane processes involving RO, FO, NF. Additionally, electrospun nanofibers as a support layer in the fabrication of TFCs have demonstrated their usefulness. However, fewer cases have been studied in which these types of membranes combining electrospinning with the TFC production method have been applied for the removal of emerging pollutants. However, the literature provides substantial information on the use of electrospun membranes in adsorption or photocatalysis, as well as other advanced oxidation processes for the retention of emerging pollutants, mostly at laboratory, pilot plant level. There is thus a need to develop studies in the direction of the application of electrospun nanofibrous membranes as a barrier layer for water treatment with the removal of emerging pollutants, with a focus on the consolidation and post-treatment of electrospun membranes.

The parameters influencing the morphology of electrospun fibers can be classified into the following groups: system, process, and environmental [34,68,96]. System parameters are focused on polymer molecular weight and solution concentration, conductivity, dielectric constant, surface tension, viscosity, and solvent type [34]. When the polymer has a high molecular weight, the formed fiber has a large diameter and is straight, without beads.

Additionally, the polymer concentration is one of the essential parameters in the electrospinning process. Low concentrations lead to jet instability and fracture, and the final product will take the form of beads. Multiple attempts are usually required to achieve a specific concentration range to form fibers of uniform diameter. Insulating polymers are difficult to electrospin, so ionic compounds or salts are used to improve the conductivity of the polymer solution [40].

If the concentration range is appropriate, the fibers are formed without defects, while a high concentration prevents the formation of straight fibers. As the electrical conductivity increases in a specific range, the diameter of the fibers decreases, and the fibers do not show pearls, while excessively increasing the conductivity values makes it impossible to form straight fibers of uniform diameter. The decrease in surface tension leads to the formation of smooth fibers, together with an increase in vascularity, the latter leading to an increase in diameter. Additionally, an increased viscosity will lead to bead-like fibers and clogging, and extremely low viscosity will lead to the electrospraying phenomenon. High solubility gives the appearance of fibers with a well-defined morphology, together with appropriate volatility [85,97].

Thus, the rheology of the polymer solution is essential in the formation of fibers, and the molecular weight and concentration of the polymer directly influence the properties of the obtained fibers. It is known that at low concentrations, when the viscosity is low, the phenomenon of “electrospraying” occurs, in which particles are formed instead of fibers [40,98]. For example, a solution of 5% poly (vinylidene fluoride) (PVDF) in a solution of *N*,*N*-dimethylformamide (DMF) leads to droplets, with smooth fibers only appearing at concentrations above 10% (wt), with diameters being of about 500 nm, while in the case of the addition of DMF/acetone, the obtained matte fibers are of about 330 nm, acetone acting to decrease the viscosity of the solution without influencing the fiber formation capacity [99].

Solvent volatility has a major impact on the resulting membrane fiber morphology. When a low-volatility solvent is used, evaporation does not occur rapidly, and thus wet fibers are formed. On the other hand, when using highly volatile solvents, the fibers solidify immediately upon exit from the needle, and the polymer jet no longer leads to the formation of fibers.

The solubility of the solvent induces a homogeneous polymer solution suitable for electrospinning [68,100,101]. The most used solvents include acetone, dimethyl sulfoxide (DMSO), chloroform, dimethylformamide (DMF), dichloromethane, tetrahydrofuran (THF), hexafluoroisopropanol (HFIP), and alcohols. Some applications involve the mixture of different solvents to produce an efficient formulation. Water is not an adequate solvent for electrospinning, due to its depletion of electrostatic repulsions, having a high dielectric constant.

The spinning voltage is the most basic processing parameter of electrospinning; it leads to the formation of the Taylor cone when a critical voltage is reached, after which, by increasing the voltage required to obtain perfectly stretched fibers, the formation of a jet responsible for fiber formation takes place [85]. The increased tension leads not only to the stretching of the fibers, but also to their thinning, with the diameter being decreased to the order of nanometers.

The speed at which the spinning solution is injected is extremely important in the formation of fibers of different diameters. Adjusting the pumping speed through the syringe can lead either to small diameters when the electrospinning period is long (when the speed is low) or to large diameters or even droplet formation at high speeds. Additionally, the distance between the syringe through which the polymer is extruded and the manifold is important in terms of solidification and fiber formation time. With a short distance, the solvent will volatilize, causing the fibers to adhere and increase in diameter. In addition, solvents with increased volatility lead to fibers wrapping around the needle, just as low volatility leads to the inability of the jet to be stretched; therefore, the use of mixed solvents to obtain ideal materials for electrospinning is recommended. As the distance increases, the formation of smaller diameters will be possible, reaching as low as nanometers.

The applied voltage influences the shape and morphology of the fibers. An example of this is the electrospinning of the mixed solution of poly (vinyl alcohol) and sodium alginate at values between 28 kV and 35 kV, for which the shape of the fibers is different [20,102].

For example, at 28 kV, broken fibers were formed, while the length of the fibers became continuous at voltages greater than 35 kV. Authors suggest that at high stresses, long, matte fibers with small diameters are typically formed [40], although there is still no exact relationship between voltage and fiber diameter, which must be correlated with the concentration of the solution and the tip–collector distance. However, it has been found that bead formation occurs at very high voltages [102].

Environmental conditions such as humidity and temperature can influence fiber morphology [103]. Thus, low relative humidity can accelerate solvent evaporation, leading to the formation of fibers with small diameters. Temperature acts in two ways on fiber morphology. As the temperature increases, the evaporation of solvent occurs rapidly, leading to difficulty in stretching the jet. When the temperature decreases, the viscosity of the solution decreases, and thus the formation of fibers with small diameter occurs.

Relative humidity (RH) influences the evaporation rate of the solvent, and thus the formation of pores on the surface of the fibers. For example, electrospinning of polystyrene (PS) fibers in tetrahydrofuran (THF) at a low humidity of 25% leads to smooth, pore-free fibers on the surface [104]. At values above 30%, more pores begin to appear on the fibers. It was thus found that the porosity and pore diameter increase with increasing moisture [40].

The effects of humidity and temperature when obtaining cellulose acetate (CA) and poly (vinylpyrrolidone) (PVP) fibers, respectively, have also been studied [105]. For CA, the fiber diameter increased with increasing humidity, while the diameter of PVP fibers decreased due to the absorption of water from the environment, which induced a slower solidification, and therefore a longer jet elongation time, resulting in the formation of fibers with thinner diameters. However, at a high relative humidity of 60%, PVP nanofibers begin to adhere to each other, thus resulting in larger apparent diameters.

In CA, with increasing RH, water absorption leads to faster precipitation, leading to the formation of larger diameters. The two materials follow opposite trends due to different interactions between the polymer/solvent system and water vapor, causing the evaporation of the solvent and the solidification rate to react differently.

**Figure 2:**
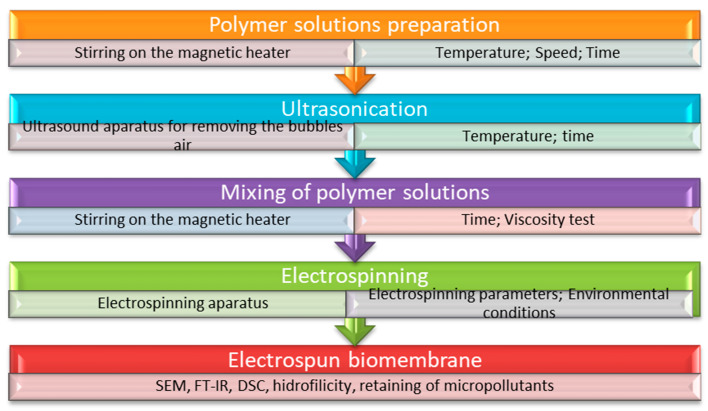
Electrospinning process steps applied for membrane fabrication.

In summary, electrospinning represents an advanced fiber preparation technology based on the interaction between a polymeric solution and an electric field, generating fibers as products [34,106]. A schematic depiction of the process is presented in Figure 2.

The electrospinning device works based on three principles that represent the operating steps [107]. (1) Extrusion of polymeric liquid from the syringe through the spinneret. Here, the droplet has a spherical shape at the tip of the spinneret due to the balance between surface tension and gravitational acceleration. (2) The morphology of the droplet changes, with the application of electric field force, from a spherical to a conical shape with increasing electric charge, a phenomenon based on gravitational acceleration and Coulomb force, which must be greater than the surface tension to generate a straight jet forming the cone. (3) According to the principle of Rayleigh instability and the interaction of positive charges on the jet surface in the electric field, the duration during which the jet is straight is short, resulting in deflection. Based on the Coulomb repulsive force and gravity, the jet in the deflection section will solidify rapidly at the same time as the solvent evaporates.

The use of electrospun membranes in the treatment of wastewater contaminated with emerging pollutants such as antibiotics has been studied intensively in recent years. Thus, it is possible to establish, on the basis of the results presented so far in the literature, specific conditions that need to be met when using electrospun membranes [34,108,109,110,111,112,113,114,115].

It is important that the polymer used to obtain the fibers to be used as membranes be environmentally friendly, biodegradable, and without secondary environmental pollution. Additionally, the obtained membranes have to possess selectivity for the target pollutants, which can be developed by functionalization with specific groups. Thus, the polymers used for electrospinning can be adsorbent, and can be used as such; additionally, substances possessing specific adsorbent functional groups can be added to the electrospinning solution, or the obtained membrane can be subjected to post-treatment techniques after the electrospinning process, such as coating [108,109], heat treatment [110,111], and cross-linking [112,113,114,115].

Other essential conditions for obtaining these electrospun membranes include water insolubility combined with hydrophilicity of the polymers, as well as good mechanical properties and chemical stability in the obtained fibers.

### 4.2. Materials

Because fibers with nanometric scale demonstrate high efficiency in wastewater treatments, we focus on their properties and structures. These types of materials can be obtained using a variety of techniques, as described by Ahmed and colleagues [40] in their review, including self-assembly and electrospinning as emerging techniques [116,117,118], drawing [119,120,121,122], template synthesis [103,123,124,125], and phase separation [126,127,128]. The type of polymer controls the morphology, shape, size, and strength of the nanofibers. Within an appropriate operation control, the obtained nanofibers exhibit porosity, favorable conditions for functionalization with nanoparticles, defect-free fibers, rigidity, and tensile strength. Electrospinning parameters, including polymer solution properties, can be changed and controlled in order to obtain different nanofiber morphologies.

**Figure 3:**
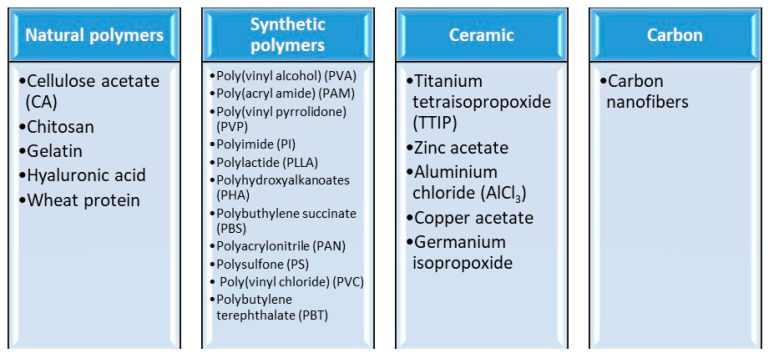
Main materials applied for the electrospinning process.

Electrospun nanofibers are excellent candidates for use as membranes in water and wastewater treatment, including dye degradation [129,130,131,132], heavy metal ion adsorption [133,134,135], oily water separation [136,137], and microbial disinfection [130,138,139]. There are many types of materials, and these are briefly presented in Figure 3.

Nanofibers exhibit new and advanced properties that can be enhanced by their functionalization. For example, due to their high specific surface area, nanoparticles, nanorods, nanowires, nanotubes, nanosheets, zeolites, and metal–organic frameworks can be immobilized, allowing the high-selectivity removal of water contaminants [140]. The functionalization can be performed through the direct electrospinning of the nanofibers using self-assembly techniques. In addition to this, nanofibers can be packed into a fixed-bed column or use membrane reactors, depending on the requirements of the separation process.

#### 4.2.1. Polymers

Usually, organic polymers are dissolved in appropriate solvents and used directly as solutions, or are melted without degradation. The advantage of using electrospun solutions is the formation of a stretched, elongated, and thinned polymer solution jet, where the solvent is evaporated, and the fibers are collected on a support [68].

Electrospun polymer solutions made from natural polymers (collagen [141,142,143,144], gelatin [145], chitosan [146,147], hyaluronic acid [148], silk fibrion [149], and/or synthetic polymers poly(lactic acid) (PLA) [150], polyurethane (PU) [151,152], poly(ε-caprolactone) (PCL) [152,153,154], poly(lactic-coglycolic acid) (PLGA) [155,156,157], poly(ethylene-co-vinylacetate) (PEVA) [158], and poly(l-lactide-co-ε-caprolactone) (PLLA-CL) [159,160] have been studied intensively. There are more than 100 polymers that can be used either individually or as a mixture; thus, the final product will mainly be a polymer fiber or a ceramic [161].

Synthetic polymers have demonstrated their high capacity for being electrospun into nanofibers, such as polystyrene (PS) and poly(vinyl chloride) (PVC), with good results in environmental protection applications. Additionally, natural biopolymers, such as silk fibroin, fibrinogens, dextran, chitin, chitosan, alginate, collagen, and/or gelatin, can be used for nanofiber preparation in various applications. Additionally, conductive polymers such as polyaniline (PANi) and polypyrrole (PPy) are able to be electrospun into nanofibers as well as poly(vinylidene fluoride) (PVDF) [68].

Among these, synthetic polymers are still used, possessing the advantages of low cost, high mechanical properties, ease of production. Some disadvantages, such as long-term health and environmental impacts due to their toxicity, nonbiodegradability, and disposal, have to be noted. In terms of waste minimization, there is an urgent need to replace them with recyclable and biodegradable green materials [140,162,163].

Additionally, some polymers that are insoluble solvents appropriate for electrospinning, such as polyethylene and polypropylene (PP), are melted and electrospun directly [164].

To be melted, a polymer has to be thermally stable (except for thermoset polymers and proteins) and non-degradable, such as thermoplastic polymers (e.g., PP) and polyesters (e.g., polyurethane, PCL, PLA, and PLGA). In the case of PCL, which has a low melting point, the thermal stability and processability are suitable for the melting electrospinning process. There are some common industrial polymers with adequate melting capacity for electrospinning: nylon-6, polyethylene, poly(methyl methacrylate) (PMMA), and poly (ethylene terephthalate) (PET). Usually, the melting process for a polymer prior to electrospinning depends on its viscosity and electrical conductivity [165].

Additionally, classes of polymers such as polyolefins and polyamides, soluble in specific solvents, are mostly processed into nanofibers using the melting electrospinning process.

CS is widely applied as a biopolymer in wastewater treatment, and its ability to be electrospun makes it a more interesting material, with a high capacity for the adsorption/degradation of different pollutants, including pharmaceutical compounds. Pure chitosan dissolved in acetic acid solvent can be used to prepare nanofibers, and subsequently membranes, for wastewater treatment [1,166]. Usually, the fiber diameter is proportionally linked with the concentration of the solution, and only 2% or less of CS is acceptable in order to obtain homogenous nanofibers with higher molecular weight, as the other polymer-processing conditions and molecular weight, together with deacetylation degree, are crucial for nanofiber processing [167].

The functionality of CS can be enhanced by mixture with other polymers and metals or metal oxides by means of amino groups [168]. For example, PEO, a synthetic polyether, was used to prepare 80:20 wt% CS/PEO solution for the removal of pharmaceuticals such as ibuprofen [169] and CS/PEO/permutit electrospun nanofibers [170]. A commonly used mixture for wastewater treatment is CS/PVA (polyvinyl alcohol), with PVA being known as a synthetic polymer that is soluble in water, and is usually employed as a film. CS/PVA nanofibers at different concentrations and temperatures were prepared using the electrospinning technique, with the tetracycline (TC) being degraded on this nanofiber [171,172,173,174,175,176,177]. Mixture blends with different compounds lead to different classes of adsorbents, such as the immobilization of ZnO into PVA/Alg/CS polymeric composite nanofibers for phenol degradation [178].

β-cyclodextrin (β-CD) is an excellent compound that is useful for the adsorption of large ions from water, and membranes prepared with electrospun β-cyclodextrin (β-CD)/chitosan/PVA nanofibers are capable of simultaneously removing organic and inorganic pollutants, the most notable of which is Bisphenol A (BPA) [179]. An important feature of these membranes is their decreasing porosity with increasing thickness. A number of CS formulations have been studied, including CS/PVA [171], CS/polyethylene oxide (PEO) [169], β-CD/CS/PVA [179], M-ZnO/PVA/Alg/CS [178], and CS-g-PNVCL/ZIF-8 [180], in order to analyze the adsorption efficiencies on pharmaceutical compounds (tetracycline, ibuprofen, Bisphenol A, and phenol).

Good adsorption capacities were obtained, with β-CD/CS/PVA nanofiber being compatible for integration as a membrane in drinking water treatments [179].

Usually, cyclodextrin derivatives induce high viscosity in solution, as it is able to form aggregates via hydrogen bonding, and possesses a viscoelastic solid-like behavior [68].

Cyclodextrin (CD) enhances the removal capacity of PAN nanofibers (91.46% for atrazine) and polyethersulfone (PES) nanofibers (for steroid hormones), and has also been investigated in combination with triglycidyl ether and triphenylmethane triglycidyl ether as crosslinkers (95% for estradiol). It was observed that when the diameter is decreased from 557 nm to 497 nm, in the case of CD-PAN, the specific surface area increases [140]. Additionally, the synergistic effect of hydrophobic interaction and hydrogen bonding influences the removal process in the case of CD-PES [181,182,183].

PVA and CD are used for the removal of emerging pollutants from wastewaters, especially when cyclodextrins (CD) act as macrocyclic hosts, and their high surface area makes them optimal substrates for the adsorption of large molecules. Additionally, PVA decorated with alkali lignin, after thermo-stabilization, offers efficient antibiotics removal [184].

Cellulose is a sustainable polymer that can be applied to obtain nanofibers [185,186,187,188,189], and has been studied intensively in recent years. In addition to the use of ionic liquids to produce cellulose fibers, the electrospinning method is also applied [185,190]. One important aspect is the defects that can appear at the nanoscale level [191]. It has been observed that low-density nanocellulose and the reactive surface of –OH groups allow the grafting of chemical species in order to acquire new functionalities as substrates for wastewater treatment [191].

Some examples of cellulose electrospun nanofibers configured as membrane systems are based on the use of amino-ionic liquids (ILs). For example, polyvinylidene fluoride-cohexafluoropropylene (PVP-HFP) nanofibers modified with cellulose using [Emim]Ac as ionic liquid possess good porosity, pore size, wettability, mechanical and thermal strength for use as nanofibers for oil separation [192]. Other examples are based on ethylcellulose nanofibrous matrix doped with ionic liquid [Emim]BF4 or electrically conductive cellulose nanofibers containing carbon nanotubes; the ionic liquid [Bmim]Cl was used to dissolve cellulose solution, for application in water desalination [185,193,194].

Cellulose acetate/cellulose triacetate (CA/CTA), along with other polymers such as polyamide, polysulphone (Psf), etc., have been used for FO membrane design [195].

There are commercial CTA membranes for application in FO that exhibit a water flux and salt rejection of 96% in comparison with RO [196]. Double-skinned CA membranes reduce fouling and exhibit high salt rejection due to their dense layer structure [197,198,199].

Various parameters influence the morphology and properties of the membrane, such as polymer–solvent concentration, evaporation and annealing time, casting substrate, coagulation time, etc. [200,201]. The functional groups play an essential role in separation efficiency, including acetyl, hydroxyl, propionyl, and butyryl groups [195].

Polysulfone (PSf) is another polymer widely applied as an electrospun substrate in the fabrication of thin-film nanocomposite membranes, especially for FO. It exhibits excellent mechanical, thermal and chemical stabilities, with the disadvantage of fouling due to its hydrophobic properties [195,202,203]. Mixture with modifiers or hydrophilic nanofillers, such as oxides (titanium and silica), can be carried out to boost hydrophilicity [204]. Furthermore, the addition of graphene to Psf leads to pH and chemical resistance due to the sulphonyl group [205].

Polyethersulfone (PES) is also known to be an electrospinnable polymer substrate, especially for FO membranes, possessing good thermal, chemical and mechanical properties, as well as pH resistance [206].

The PES membrane increases the porosity, and improves permeability and tensile strength, and mixture with carbon nanotubes was demonstrated to be efficient for seawater desalination and wastewater treatment [195].

There are various polymers that are included in membrane system configurations, and their electrospinnability properties are used in order to obtain controlled nanostructures. Thus, the polymers act as a substrate both for TFC membranes (used in membrane separation processes) and for the integration of other active compounds (used in adsorption and degradation/advanced oxidation processes).

#### 4.2.2. Composites Using Carrier Polymers as Substrate

Nano or colloidal particles, as well as other small molecules, can be electrospun into fibers with the help of polymer solutions as carriers. The process is sustained by adequate intramolecular interactions when self-assembled structures appear [68].

Composites are prepared by adding sol−gel precursors or other nanoparticles into polymer electrospinnable solutions, where the polymer has a “carrier” function. The electrospinning process is linked to the characteristics of the sol−gel precursor and the carrier polymer, in order to obtain adequate composite nanofibers [68].

An inorganic phase is built up as a continuous network in the polymer matrix, and the formation of inorganic−polymer composite nanofibers takes place. PVP is one of the polymers most widely used as a carrier polymer due to its high solubility in ethanol and water and good affinity for different sol−gel precursors. In addition to this, PEO, poly(vinyl alcohol) (PVA), and poly(acrylic acid) are also used [207,208,209]. The viscosity and electrical conductivity of the solution are important characteristics for obtaining an electrospinnable solution, and the carrier polymer has to expose high Mw [68,210]. Salts such as NaCl and (CH_3_)_4_NCl) ensure electrical conductivity of the solution and determine whether thin fibers are obtained. For example, nanofibers from PVP and amorphous TiO_2_ were produced from a mixture of PVP and titanium tetraisopropoxide (Ti(OiPr)_4_, as a precursor for TiO_2_, dissolved in alcohol [207]. The sol−gel precursor reaction (hydrolysis, condensation, and gelation) has to begin when the electrospinning jet reaches the surrounding air [68,207,208]. The sol–gel reactions are influenced by precursor type; thus, rapid hydrolysis results in the clogging of the spinneret, and a quick gelation creates a non-flexible jet and thicker fibers [208]. Precursors such as alkoxides, nitrates, acetates, chlorides, and sulfates are intensively used, and in order to ensure a proper hydrolysis and/or gelation process, additives such as acetic acid, hydrochloric acid, or propionic acid can be added [211,212]. Additionally, composites were prepared by dispersing nanoscale components into polymer solutions through stirring or ultrasonication. Some well-known examples include Ag, Au, TiO_2_, and SiO_2_ nanoparticles, carbon nanotubes, clay tablets or metal−organic framework (MOF) compounds [213]. The electrospinning process depends on the morphology, size and concentration of the nanocomponents. It is necessary to ensure a stable dispersion in the polymer solution of the nanoscale components in order to obtain homogeneous nanofibers. Ag nanoparticles can be well dispersed in an aqueous PVA solution or PVP to obtain controlled composite nanofibers, and SiO_2_ particles dispersed in PVA solution fibers composite fibers with a necklace-like structure [213,214,215].

TiO_2_ nanofibers are deposited onto a polyvinylidene difluoride (PVDF) membrane as a support. This type of material, as a composite, was tested for the degradation of emerging pollutants. Good results were achieved for BPA degradation using a UV light source, with an efficiency of about 63–85% [140,216]. Additionally, the electrospinning method was applied for the preparation of ZnO–carbon composite nanofibers, using PAN, polystyrene (PS) and polyvinyl pyrrolidone (PVP) as precursors for caffeine (80.4%) and diclofenac (79.5%) removal under photocatalytic degradation [217]. A TiO_2_ nanofiber layer with a thickness of between 10 and 29 µm was embedded on a stainless-steel filter, with PVDF in between as a binder, was tested, and demonstrated high efficiency for cimetidine (90%) [218]. TiO_2_ photocatalysts are classified into two arrangements, suspended and immobilized in/on the support, with higher photocatalytic capacity for absorbing more UV light in the case of suspended TiO_2_ due to the high surface area in contact with pollutants, but with the disadvantage of a low recovery rate from solution. Satisfactory results were obtained for photocatalytic oxidation of BPA (84.53%) using a PVDF/TiO_2_ nanocomposite membrane with a uniform structure [216]. The composite membrane presents a porous-like structure, and TiO_2_ was immobilized on a PVDF substrate.

Numerous emerging contaminants were evaluated as antibiotics in terms of their degradation efficiency (over 90%) when PAN nanofibers with TiO_2_ nanoparticles dispersed in the polymeric matrix were applied as nanofiber composite filters [140,219]. The dispersant of the nanoparticles was phthalic acid, which increased the diameter of the nanofibers and their porosity by means of high viscosity and volatilization rate.

TiO_2_ nanoparticles can be added to the polymer solution of PS in order to obtain a hydrophilic electrospun membrane. This structure is then coated with nano g-C_3_N_4_ using melamine as the precursor, and a core–shell CNCT functional nanofiber membrane is obtained for TC and Escherichia coli degradation [34,220]. Additionally, a high concentration of metal nanoparticles dispersed in polymer solution results in a spinnable mixture. Ag nanoparticles were attached to the surface of PAN fibers, using AgNO_3_ precursor solution and irradiation of the fibers under a UV lamp to reduce Ag+ to Ag nanoparticles [221].

Colloidal particles can be electrospun when inorganic sols are the result of the hydrolysis and condensation of metal alkoxides, or when metal salts possess viscosity. A silica sol obtained from tetraethyl orthosilicate (TEOS) as a precursor generates silica fibers with sizes between 0.4 and 1 μm or smaller than 400 nm, depending on the working parameters [68,222].

Ag nanoparticles were directly dispersed in ethylene glycol, and after thermal annealing, conductive Ag nanofibers were obtained [68].

The electrospinning method was also applied for hollow and porous Fe-doped PAN nanofibers developed for the removal of BPA. The nanoparticles were uniformly immobilized, and 100% BPA removal was achieved [140,223]. To degrade the emerging pollutants, nanofiber membranes can be used in electrochemical processes. For example, antimony tin oxide doped with ruthenium oxide (ATO-RO) nanoparticles was integrated into PVP nanofibers. The material acts as the anode for electrochemical BPA degradation [224]. Additionally, TC solutions were subjected to degradation using Fe/Co alloy on PVP nanofibers, with efficiencies of up to 100%. Efficiencies over 95% were registered when tubular carbon nanofibers with activated alumina were integrated into a PVC support and used as the anode material for the electrocoagulation process [225,226].

Electrospun composites based on carbon were recently developed for the adsorption of antibiotics. Carbon nanofiber (CNF)–carbon nanotube (CNT) composites, with high specific surface area and mechanical strength due to the nanofibers, exhibited high uptake capacity (>90%) and fast kinetics [140,227].

### 4.3. Performances of Electrospun Materials Used for Adsorption and Advanced Degradation Processes

The literature already offers solutions, in addition to existing membrane systems, of combined methods for the retention of emerging pollutants using electrospun membrane structures, decorated with nanoparticles with adsorbent or photocatalyst role [140]. However, a classification according to the type of category or emerging pollutant, compared to other types of membrane systems, has not been made. Nanofibers act as adsorbents for emerging pollutants. Through their structure and appearance, they can be designed to incorporate target compounds with advanced properties due to their functional groups, and their high specific surface area, which possesses a high selectivity that subsequently acts on the degradation process of pollutants. Thus, once immobilized on the nanofiber surface, emerging pollutants can be degraded by advanced oxidation, or catalytic or electrochemical degradation processes. Nanofibers can act as a carrier for reactive nanoparticles with high degradation potential, but which, when used alone in the system, may have a tendency to agglomerate, reducing their effect, or which, due to their tendency to disperse in aqueous media, can pass through classical filtration systems into natural systems, their size making them difficult to collect from waters. Thus, their incorporation into nanofibers leads to the development of adsorption systems and the simultaneous degradation of emerging pollutants. Another important aspect is the possibility of regenerating these functionalized nanofibers and using them for multiple cycles.

Additionally, as was mentioned, electrospun fibers can be integrated into TFC as support due to their mechanical properties and flexibility, and the electrospun structures (micro- and/or nano-dimensional in size) are responsible for membrane separation phenomena, in addition to adsorption or photo- and electro-catalytic degradation [40].

In summary, the most significant decontamination processes in which nanofibers have been tested are presented in Table 2, with a focus on the main EP types and categories.

### 4.4. Performances of Electrospun Materials Integrated into Separation Membranes Processes

There are many types of membranes used in the separation (filtration) or adsorption retention of emerging pollutants. Among these, the type of membrane used in these processes is very important, as they must meet certain characteristics regarding pore size, active surface, permeability, material type. Thus, today, a variety of materials based on polymers, ceramics, zeolites, etc., are known to be used in the manufacture of membranes. Ultrafiltration (UF), microfiltration (MF), nanofiltration (NF), reverse osmosis (RO), and forward osmosis (FO) are defined in the literature as processes that are dependent on pore size, morphology, and specific characteristics of the type of water and pollutants [2]. In Table 3, the most significant materials integrated into membrane processes are presented.

It can be seen that membranes are an effective means of reducing emerging pollutants during both drinking water and wastewater treatment. The degree of removal is directly related to the characteristics of the membrane and, to some extent, to the molecular properties of the contaminant in question [271].

As a rule, microfiltration/ultrafiltration (MF/UF) systems are recommended primarily due to space limitations, and show different efficiencies depending on the class of emerging pollutants. As a rule, for organic compounds, performance is limited unless coupled with RO, but there are promising results in the removal of steroids.

Nanofiltration and reverse osmosis are effective for the removal of emerging pollutants although some compounds could be detected in traces in the permeate. In addition, continuing concerns about possible quality of life impacts of emerging pollutants require viable solutions to be found and tested, and these techniques are proving effective.

## 5. Conclusions

The implementation of different functional membranes in water treatment is greatly increasing, with a focus on emerging pollutants.

The most used materials applied in membrane design are polymers, both as support for TFC, with application in separation processes, and also as functional materials applied in adsorption and other advanced processes. Electrospinning represents a facile and adequate method for fiber preparation (especially nanosized), where surface characteristics and functionalization enhance material properties. Using electrospun fibers, various emerging pollutants can be degraded, especially pharmaceutical compounds. Some reliable results have been reported on the use of electrospun membranes for separation processes in pilot and/or full-scale applications. An significant number of efficient electrospun nanofibers have been investigated in the literature in systems testing adsorption, advanced photodegradation, and other advanced processes. Good characteristics of the fibers include their reusability and their controlled surface area.

Due to their performances, an integrated holistic strategy could be developed at an industrial level in order to control and preserve the aquatic environment when emerging pollutants threaten the ecosystem’s equilibrium. Setting new standards for the quality of wastewater treatment is dependent on material performances that contribute to water management systems. Research should be focused on the development of hybrid systems for degradation and removal of emerging pollutants from wastewaters.

## Figures and Tables

**Table 1:** Advantages and future challenges for applying conventional and non-conventional processes for EP removal.

Water Treatment Technologies for the Removal of Emerging Pollutents (EP_S_)			
Conventional Processes		Non-Conventional Processes	
**Biological Processes**			
**Activated sludge**		**Constructed wetlands**	
**Advantages**	**Challenges**	**Advantages**	**Challenges**
- Greener than chlorination - Lower operational costs than AOPs	- Low efficiencies for pharmaceuticals and beta blockers - Large amount of sludge containing EPs	- Low energy consumption, low operational and maintenance costs - High removal efficiency for estrogens and pathogens	- Clogging, solids entrapment, sediments formation - Seasonal dependent, chemical precipitation, biofilm growth - Large areas of land needed and long retention time
**Biological activated carbon**		**Membrane reactor systems (MBR)**	
**Advantages**	**Challenges**	**Advantages**	**Challenges**
- Removal of a broad range of EPs - Removal of residual disinfection/oxidation products - No generation of toxic active products	- High operation and maintenance costs - Regeneration and disposal of high sludge amounts that increase total costs by 50–60%	- Effective for the removal of biorecalcitrant EPs - Small footprint	- High energy consumption and fouling control of heat and mass transfer - High aeration costs and roughness of membrane - Low efficiencies for pharmaceutical pollutants
**Microalgae reactor**			
**Advantages**	**Challenges**		
- Resource recovery from algal biomass used as fertilizer - Efficient effluent and no risk of acute toxicity	- Removal efficiencies affected by cold season - EPs not degraded properly		
**Chemical Processes**			
**AOPs**		**Waste Stabilization Ponds (WSP)**	
**Advantages**	**Challenges**	**Advantages**	**Challenges**
- Short degradation rate	- High energy, operational and maintenance costs - Toxic disinfection by-products - Interference of radical scavengers	- Minimum cost, simple and better for pathogen elimination - Low operational and maintenance costs	- High energy consumption and fouling control of heat and mass transfer - High aeration costs and roughness of membrane - Low efficiencies for pharmaceutical pollutants
**Coagulation**		**Oxidation Ditches (OD)**	
**Advantages**	**Challenges**	**Advantages**	**Challenges**
- Reduced turbidity due to suspended inorganic and organic particles - Increased sedimentation rate through suspended solid particle formation	- Ineffective micropollutant removal - Large amount of sludge - Introduction of coagulant slats in the aqueous phase	- Low operational and maintenance cost	- Tertiary filters may be prerequisite after elucidation, reliant on the sewage necessities
**Fenton and photo-Fenton**			
**Advantages**	**Challenges**		
- Degradation and mineralization of EPs	- Formation of chloro and sulfato-Fe(III) complexes, in the presence of chloride and sulphate ions		
**Ozonation**			
**Advantages**	**Challenges**		
- Strong affinity to EPs in the presence of H_2_O_2_ - Selective oxidant favoring disinfection and sterilization properties	- High energy consumption, formation of oxidative by-products		
**Photocatalysis (TiO_2_)**			
**Advantages**	**Challenges**		
- Sunlight can be used - Degrading persistent organic compounds - High reaction rates upon using catalyst - Low price and chemical stability of TiO_2_ catalyst and easier recovery	- Difficult to treat large volume of wastewater - Cost associated with artificial UV lamps and electricity - Hard to separate and reuse from slurry suspension		
**Physical Process**			
**Micro- or ultra-filtration**		**Aeration systems with new types of membranes**	
**Advantages**	**Challenges**	**Advantages**	**Challenges**
- Can remove EPs and pathogens	- Not efficient in removing some large EPs	- Significant reduction of load losses of up to max. 80 mbar (as opposed to conventional systems); - Membrane resistance temperature of approx. 80 °C; - Minimum system maintenance costs - Low energy consumption	- Limited testing and upscale implementation data
**Nanofiltration**			
**Advantages**	**Challenges**		
- Treating saline water and wastewater treatment plants (WWTP) influents - Can remove dye stuff and pesticides	- High energy demand, disposal issues - Limited application in pharmaceuticals removal		
**Reverse osmosis**			
**Advantages**	**Challenges**		
- Treating saline water and WWTP influents - Can remove PCPs, endocrine disrupting compounds (EDCs) and pharmaceuticals	- High energy demand, disposal issues - Corrosive nature of finished water and lower pharmaceutical removal		

**Table 2:** Examples of nanofibers with efficiencies in adsorption and advanced decontamination processes for EP.

Type of Process	Type of Nanofiber/Method/Characteristics	EP Types/Category, Performances
Adsorption [184]	Anionic nanofibrous nonwoven adsorbent: alkali lignin and poly(vinyl alcohol) (PVA). Method: 4 h thermal treatment (180 °C), 120 min chemical treatment (citrate buffer solution 0.5 M, pH 4.5). Diameter: 156 nm.	Pharmaceutical contaminant (32 ppm fluoxetine), contact time 1 h, Adsorption efficiency: 70%.
Adsorption [181]	Electrospun PAN nanofiber membranes modified with β-cyclodextrin (β-CD) crosslinked with citric acid. Method: PAN and PAN-CD (ratio 80:20) prepared in DMF solution, 12 h at room temperature. Citric acid (0.1 M) as crosslinker and sulfuric acid (0.05 M) as activator. Diameter: 557 nm PAN and 497 nm PAN with β-CD.	Atrazine (5–25 ppm), adsorption capacity: PAN 0.603 mg/g, PAN-CD 0.817 mg/g. Adsorption efficiency: PAN 67% and PAN-CD 91%.
Adsorption [182]	Porous β-cyclodextrin modified cellulose nano-fiber membrane (CA-P-CDP). Method: prepared PCDP was dispersed in a mixture of prepared CA membrane and NaOH solution, 2 h. Freeze-drying for 24 h. Diameter: 462 ± 94 nm for nanofibers of CA membrane.	Bisphenol A (BPA), S (BPS), F (BPF): 1 mg/L, adsorption capacities (15 min): 50.37, 48.52, 47.25 mg/g.
Adsorption [183]	Composite nanofiber membrane (CNM). Method: polymerization of βCD using epichlorohydrin (EP) and deposited β-cyclodextrin-epichlorohydrin (βCDP) on PES ultrafiltration (UF) membranes via electrospinning. Diameter: 90–250 nm for surface of CNMs, cross Section 250 thickness of about 80 μm.	Radiolabeled steroid hormones. Removal efficiency estradiol E2 (2.59 TBq/mM): 80% (5 h) static adsorption, and 99% dynamic filtration.
Adsorption [171]	chitosan/poly (vinyl alcohol) glutaraldehyde-crosslinked electrospun nanofibers (GCCPN). Minimum diameters: 6–18 nm, 75/25 chitosan/PVA ratio.	50–250 mg/L TC. Maximum adsorption: 102 mg/g. Adsorption efficiency: 34–97%.
Adsorption [228]	Polyporous electrospun fibrous membranes via electrospinning: methoxy polyethylene glycol-poly(lactide-co-glycolide) (MPEG-PLGA), poly(D,L-lactide-co-glycolide) (PLGA) and poly(D,L-lactide) (PDLLA). Triblock copolymer/polymers/solvent weight ratio: 1/10/90, 1.5/15/85 and 2/20/80, dissolved in methylene dichloride, vigorous stirring. Diameters: 740, 530 and 470 nm for MPEG-PLGA, PLGA, and PDLLA.	10 g/L triclosan (TCS). Maximum adsorption capacities MPEG-PLGA, PLGA and PDLLA: 130, 93 and 99 mg/g. Removal efficiency: over 90% with decreasing at 80% in case of competitive adsorption.
Adsorption [229]	Fiber-adsorbent from cellulose acetate (CA) membrane via electrospinning. Method: homogeneous CA solution from cellulose acetate added to 4:1 chloroform/methanol mixture, stirring and sonication. Adding under vigorous stirring of 1-Butyl-3-methylimidazolium hexafluorophosphate (BMIPF6) used as ionic liquid to obtain a homogeneous CA-BMIPF6 solution as precursor. Diameters: 100−400 nm, more than 10 cm long. Average pores diameter: 3 nm.	25 mg/L Triclosan (TCS), Adsorption capacity: 797.7 mg/g.
Adsorption [230]	Fiber membrane with interconnected mesopores based on an electrospun zeolitic imidazolate framework-8 (ZIF-8)/PAN fibers integrated into PVP. Method: zinc salt and 2-methylimidazole as precursors into PVP to obtain electrospun fiber membrane, PVP removal at 50 °C for 24 h, membrane soaked into methanol 3 days, dried at 100 °C in vacuum. Diameters: 36–112 nm.	TC, maximum adsorption capacity: 885.24 mg/g, after 4 h. Adsorption efficiency 97% after 10 cycles.
Adsorption [231]	Alkali lignin AL and poly (vinyl alcohol) PVA nanofibers. Method: mixing 2 solutions: AL dissolution in NaOH 1 M (1) and PVA in water (2), heated to 80 °C, 60 min. Mass ratio of 1:1 of (1): (2) for electrospinning, refrigerated 4 °C max 1 month. Electrospun fiber stabilization: heating at 160 °C, 3 h, membranes immersion into sodium citrate buffer pH 4.5, 3 h. Diameters: 183 ± 5 nm by electrospinning, 156 ± 5 nm by thermal process, 188 ± 10 nm by chemical stabilization.	Fluoxetine (FLX), venlafaxine (VEN), carbamazepine (CAR), ibuprofen (IBU). Individual adsorption: FLX: 78.24 ±1.35 mg/g (78%), VEN: 49.76 ± 2.80, CAR: 8.04 ± 0.01, IBU: 5.00 ± 0.46 mg/g. Desorption tests: 90% recovery.
Adsorbtion [232]	4 types of nanofiber mats metalorganic frameworks (MOFs): polydopamine (PDA) modified electrospun PVA/SiO_2_ as organic inorganic hybrid nanofiber. Method: electrospun PVA/SiO_2_ nanofibers immersed in PDA 12 h, autoclaved with ionic liquids: MIL-53(Al), Uio-66-NH_2_ and NH_2_-MIL-125(Ti). Deposition efficiency: MIL-53(Al) > NH_2_-MIL-125(Ti) > UiO-66-NH_2_ > ZIF-8. Diameters: 0.3–0.5 mm thick for PVA/SiO_2_ nanofiber mat, >1000 nm for 3D-PDA-modified PVA/SiO_2_ nanofibers.	Chloramphenicol (CAP), equilibrium adsorption capacities: ZIF-8 (13.9 mg/g) < UiO-66-NH_2_ (25.1 mg/g) < NH_2_-MIL-125(Ti) (49.5 mg/g) < MIL-53(Al) (79.5 mg/g).
Adsorption [233]	Fe_3_O_4_/polyacrylonitrile (PAN) composite nanofibers. Method: two-step process: electrospinning (8 h) and solvothermal method. The fibrous mat collected after electrospinning cut to 5 cm × 2 cm, immersed in FeCl3 dissolved in DEG, added Na_3_Cit and anhydrous sodium acetate, 80 °C, autoclave. Average diameter: 500 nm (single NF), 60 nm (Fe_3_O_4_ NPs), 20 nm (coating thickness).	TC. Maximum adsorption capacity (Langmuir isotherm): 257.07 mg/g, pH 6. 5 cycles of adsorption-desorption.
Adsorption [233]	polyimide (PI)-based carbon nanofibers (CNFs). Method: electrospining polyamic acid solutions, thermal imidization and carbonization. Polyamic acid PAA nanofibers dried overnight, imidization of PAA fibers and carbonization at different temperatures and time intervals. High specific surface area: 715.89 m^2^/g.	2,4-DCP and TC, different temperatures. Maximum adsorption: 483.09 mg/g (2,4-DCP), 146.63 mg/g (TC). Desorption: 5 consecutive cycles.
Adsorption [234]	Zeolitic imidazolate framework-8 (ZIF-8) functionalized composite electrospun fiber. Method: adsorbent polydopamine (PDA) onto the surface of PAN electrospun nanofibers (PDA/PAN). PDA/PAN fibers immersed in Zn (NO_3_)_2_ solution 1 h, room temperature, adding 2-methylimidazole solution, heated, 40 min (ZIF-8 crystals onto fiber surface), washed and dried overnight. Average diameter: 349 nm.	TC: 478.18 mg/g, adsorption efficiency: 85%. 5 five adsorption/desorption cycles.
Adsorption [235]	Electrospun montmorillonite-impregnated cellulose acetate nanofiber membranes (MMT-CA-NFM). Method: fine powder MMT onto CA nanofibers, with acetone and dimethyl acetamide as solvents, stirring, 2 days. Diameters: 24–41 nm.	Ciprofloxacin (CIP). Adsorption efficiency: 76% pH 6. Maximum adsorption capacity: 13.8 mg/g. Reusability capacity.
Adsorption [236]	Graphene oxide (GO)/poly(vinylidene fluoride) (PVDF) electrospun nanofibrous membranes (ENMs). Method: GO-PVDF blend solution from mixture of PVDF in *N*,*N*-Dimethylformamide (DMF) and acetone with GO, deposited on aluminum foil. Average diameter: 161.67 ± 61.5 nm.	5–500 mg/L TC The maximum TC adsorption capacity of GO is 720.26 mg/g. The maximum experimental TC removal capacity (qa,exp) was 17.92 mg/g with 1.5 wt% of GO (GO1.5/PVDF) ENMs.
Adsorption [237]	Polyimide modified carbon nanofibers composites. Method: electrospinning, facile hydrothermal process and carbonization. β-cyclodextrin (β-CD) as carbon precursor for hydrothermal carbon nanoparticles (HTCNPs) and PI (polyimide) fibers as support scaffold for HTCNPs via hydrothermal process, carbonization under nitrogen atmosphere. Diameters: 2–10 nm (mesoporus).	TC, maximum adsorption capacities: 543.48 mg/g, removal efficiency: 82.32%. The basic fiber skeleton of porous structure maintained for 5 consecutive cycles.
Adsorption [238]	Carbon nanofibers (CNFs). Method: PAN polymer solutions in *N*,*N*-dimethylformamide (DMF), stirring 3 h, 75 °C, via electrospinning and thermal treatment. Fibers carbonized at 900 °C, 1 h, dried at 110 C, 24 h. Average diameter: 500 nm.	CIP, BPA, 2-chlorophenol (2-CP). Maximum adsorption capacities: 2-CP (6.18 mmol/g) > BPA (4.82 mmol/g) > CIP (0.68 mmol/g).
Adsorption [239]	Electrospun PVA fibers. Method: Mondia whitei polymeric extract frozen at −80 °C, dried, blended with PVA at different ratios, dissolved in formic acid, stirring, 60 °C, 2 h. Average diameter: 99 ± 0.023 nm.	0.5–1.25 mg/L for each 13 antiretrovirals and related drugs from wastewater (influent and effluent). Maximum adsorption capacity: 75–320 mg/g. The removal efficiency after spiking 25 mL of the real wastewater sample (effluent and influent) with 10 mg/L of standard mixture solutions.
Adsorption [140,184]	PVA nanofibers. Methoad: lkali lignin (AL) and PVA solutions (50:50).	Fluoxetine, removal efficiency: 70%.
Adsorption [227]	Carbon nanofiber (CNF)–carbon nanotube (CNT) composite based on PAN polymer solution via electrospinning and carbonization.	10 CECs (atrazine, sulfamethoxazole etc.). Removal efficiency > 90%.
Adsorption [181]	PAN–CD nanofibers (PAN nanofiber modified with cyclodextrin). Diameters: 497 nm.	10 mg/L atrazine. Removal efficiency: 91.46%.
Adsorption [182]	Cellulose nanofibers incorporating CD.	(BPA), bisphenol S (BPS), and bisphenol F (BPF). Maximum adsorption capacities: 50.37 mg/g (BPA), 48.52 mg/g (BPS), 47.25 mg/g (BPF), pH 7. 5 cycles adsorption-desorption.
Adsorption [183,240]	UF membrane. Method: electrospinning for polyethersulfone (PES) nanofibers preparation with CD deposited over PES, with different crosslinking agents (epichlorohydrin, trimethylolpropane, etc.).	Steroid hormones. Removal efficiency: 95%, estradiol, 5 h.
Adsorption [218]	PVDF photocatalytic stainless-steel filter. Method: hot-pressed TiO_2_ nanofibers over metal filter with PVDF as binder.	Cimetidine. Removal efficiency: 90% for 29 µm thickness.
Adsorption [219]	PAN nanofibers dopped with TiO_2_ nanoparticles. Method: TiO_2_ NPs dispersed in polymeric matrix with phthalic acid as dispersant.	0.5 µM CECs (atrazine, benzotriazole, caffeine, carbamazepine, metoprolol, naproxen, sulfamethoxazole). Efficiency: 90%.
Adsorption with oxidation [223]	Hollow and porous Fe-doped PAN nanofibers. Method: electrospinning and thermal treatment, activating with peroxymonosulfate (PMS).	BPA. Adsorption and oxidation efficiency: 100%, 6 min.
Degradation [225]	PVP Fe/Co alloy on PVP nanofibers.	TC, degradations: 100%, 93.12%, 88.38% at 30 mg/L, 40 mg/L, 50 mg/L.
Degradation [39]	Nanofiber Photocatalyst. Method: disperse graphitic carbon nitride (g-C_3_N_4_) into recycled polyethylene terephthalate (PET) solution, electrospinning and hydrothermal treatment. Diameters: 3.7 nm thickness for as-prepared g-C_3_N_4_.	2 × 10^−5^ mol/L Sulfaquinoxaline (SQX), sulfadiazine (SD), sulfamerazine (SMZ). Degradation rate: 100% SQX, solar irradiation, 2.5 h and about 98% for SD and SMZ at different solar irradiation times.
Degradation [223]	Porous and hollow one-dimension Fe/N-doped carbon nanofibers (Fe/NCNFs-9). Method: immobilizing Fe-MIL-101 on PAN nanofibers (Fe-MIL-101@PAN) via electrospinning, 900 °C carbonizing. Diameter: Fe-MIL-101: 530 nm.	20 mg/L BPA completely degraded with PMS peroxymonosulfate (0.2 g/L) as activator and Fe/NCNFs-9 (0.4 g/L) within 6 min.
Antibacterial degradation [241]	PSf/TiO_2_/AgNPs nanocomposite substrates as FO membrane. Method: TiO_2_/AgNP nanocomposite particles using dopamine hydrochloride (DOPA), dispersion with polysulfone PSf, electrospuned on PET nonwoven scaffold.	Tetracycline-resistant genes (TRGs). The rejection under AL-FS (active layer-facing feed solution) and AL-DS (active layer-facing draw solution): 28.53% and 24.48%.
Electrochemical degradation [224]	ATO/RO composite nanofibers as dimensionally anodes. Method: RuO_2_ (RO) as primary electrocatalyst with Sb-doped SnO_2_ (ATO) as support material via dual nozzle electrospinning. Fiber mats preparation: 200 C for 2 h, and 475 °C for 12 h. Avg diameter: 172 nm, primary nanoparticles: 10–30 nm.	0.25 mM BPA, degradation with current density of 3 mA·cm^−2^ and ATO/RO (30:1): 100%.
Electrochemical degradation [225]	Electrospun composite nanofibers base on iron/cobalt alloy nanoparticles (Fe/Co-CNFs) integrated into PVP. Method: 5.0 wt% of ferric and cobalt nitrate as precursor, direct calcination of PVP composite nanofibers, 800 °C, 30 min, reduction atmosphere.	TC, degradation: 97.55%, after 10 cycles of electrocatalytic process, 1.0 V (vs. SCE) voltage, pH 5.0, 0.1 mol L^−1^ Na_2_SO_4_ as electrolyte.
Electro-Fenton catalyst [242]	Electrospun three-dimensional (3D) nanofiber network. Method: water-resistant 3D PVA nanofiber network preparation from PVA/urea solution, crosslinked in ethanol solution containing glutaraldehyde and HCl. Spongy zero-valent iron (ZVI) preparation: Fe(III) ions reduced complexed with 3D PVA nanofiber network using NaBH_4_ solution, washed, frozen 2 h.	Sulfathiazole (STZ). Coupled adsorption and electro-catalytic oxidation rate: almost 100%, 5 min. 3D-E-Fenton experiments: 50% STZ adsorption, and total adsorption at 240 min.
Electrochemical oxidation [224]	Antimony tin oxide doped ruthenium oxide (ATO-RO) nanoparticles incorporated into PVP nanofibers via electrospinning for nanofiber used as anode material for electrochemical oxidation.	0.25 mM BPA, complete degradation, 20 min electrolysis at 3 mA/cm^2^ current density.
Electrocoagulation [226]	PVC tubular carbon nanofibers with activated alumina over PVC support as the anode material for an electrocoagulation system.	caffeine, sulfamethoxazole, acetaminophen. Degradation efficiencies: 95.8%, 94.9%, 79.8%.
Photodegradation [243]	8.4 wt% TiO_2_ coaxial nanofibers using PVA as carrier polymer.	Isoproturon. 38% photocatalytic activity.
Photodegradation [216]	PVDF/TiO_2_ Nanocomposite membrane: electrospun titanium dioxide (TiO_2_) nanofibers onto PVDF flat sheet membrane. Method: hot press technique at 100 °C, 160 °C and 180 °C for 30 min. Photocatalyst TiO_2_ nanofibers are stabilized onto PVDF membrane as support.	10 ppm BPA aqueous solution. Degradation efficiency: 63–85%, under UV radiation.
Photodegradation [217,244]	ZnO-Carbon composite nanofibers. Method: different precursor polymers solutions (PAN, PS, PVP) dissolved in DMF, addition of 8 wt% Zn(acac_)2._ Final products: 1D ZnO-X nf (X: PAN, PS or PVP).	30 ppm Caffeine (pharmaceutical drug). Degradation efficiency: 80% for 1D ZnO-PS nanofiber.
Photodegradation and oxidation [218]	Photo-catalytical active stainless-steel filter (P-SSF). Method: electrospun TiO_2_ nanofibers integrated onto SSF surface through hot-press process, using poly (vinylidene fluoride) (PVDF) nanofibers interlayer as binder. Thickness: electrospinning 0.75, 1.5, 2.25, 3.0, and 5.0 mL PVDF solution for 12, 22, 32, 42, and 64 µm. PVDF NF Diameters: 0.15–0.78 µm.	Pharmaceuticals. Cimetidine degradation: 90%, at 10 L/m^2^ h and 0.1–0.2 kPa. TiO_2_ NFs thickness from 10 to 29 µm with oxidation of cimetidine from 42% to 90%. Degradation: cimetidine > propranolol > acetaminophen > sulfamethoxazole.
Photodegradation [219]	Carbon/TiO_2_ (C/TiO_2_) nanofiber composite filters. Method: PAN nanofibers with embedded titanium dioxide (TiO_2_) nanoparticles via electrospinning, carbonization. Filter thickness: 300–1800 μm.	0.5 μM for each 8 organic micropollutants (atrazine, benzotriazole, caffeine, carbamazepine, DEET, metoprolol, naproxen, and sulfamethoxazole). Degradation: 40–90%, for 300 μm thick filter.
Photodegradation [245]	Porous nanofibers (g-C_3_N_4_@PET). Method: polyethylene glycol (PEG) and polyethylene terephthalate (PET), and graphitic carbon nitride (g-C_3_N_4_) via electrospinning, post-processing for PEG removal. Diameters: 2–50 nm.	sulfaquinoxaline (SQX), sulfachloropyridazine, sulfamerazine, sulfadiazine, sulfamethoxydiazine, p-benzoquinone, p-chlorophenol. Degradation: 90%. SQX: 10 consecutive cycles.
Photodegradation [216]	Photocatalysts membrane. Method: PVDF as support for hot-pressed TiO_2_ nanofibers.	10 mg/L BPA, Degradation efficicncy: 63–85%, UV light.
Photodegradation [217]	ZnO–carbon composite nanofibers. Method: electrospinning with different polymeric precursors (PAN, PS, and PVP), carbon doping efficiency depend on the precursors.	Caffeine, diclofenac. Degardation rate: 80.4%, 2 h for caffeine, 79.5% for diclofenac.
Adanced Photodegradation coupled with H_2_O_2_ [241]	Polylactic acid (PLA)/TiO_2_ hybrid nanofibers deposited on fiberglass supports. Method: TiO_2_ nanoparticles added to the PLA solution mixed with acetone/DMF (3:2 ratio), 60 °C, 600 rpm, 4 h. TiO_2_/PLA solution electrospun onto PLA surface as adhesive between nanofibers and fiberglass surface.	300 mg/L Ampicillin, pH 3 with peroxide, 2 cycles. Complete degradation. Limitation: degradation of PLA under the photocatalytic conditions.
Antibacterial Photodegradation [220]	Soft and heterostructured g-C_3_N_4_@Co-TiO_2_ (CNCT) nanofibrous membranes. Method: electrospinning and thermal polymerization process for Co-TiO_2_ nanofiber: PVP ethanol solution with TiO_2_ sol (1/1 ratio), stirred 1 h, electrospinning, fibrous membranes obtained calcined at 600 °C, 60 min, air. TiO_2_ sol preparation: mixture of TIP, Co(NO_3_)_2_·6H_2_O, EtOH, and HAc (1/0.03/3/3 ratio). In situ synthesized g-C_3_N_4_ nanoshell wrapped onto Co-TiO_2_ nanofiber as core-shell quantum heterojunction. Diameters: 305 nm Co-TiO_2_, 320 and 338 nm for CNCT-3 and CNCT-5 membranes (different melamine content).	Antibiotics (20 mg/L, pH 7): tetracycline hydrochloride (TC-H), doxycycline hydrochloride (DC-H), oxytetracycline hydrochloride (OTC-H), CIP. Degradation efficiency: 82.3 (CNCT-1), 90.8 (CNCT-3), and 75.7% (CNCT-5) for TC-H, 60 min. 60.2, 75.3, 82.2% for CIP, OTC-H, DC-H, visible light, 60 min.

**Table 3:** Membrane materials used in EP separation processes.

Type of Process	Membrane Material	EP Type/Category, Source	Performances/Limitations
MF [2,246,247,248]	polyether sulfone (PES), cellulose acetate (CA), nitrocellulose, polyester, regenerated cellulose, polyamide.	0.2 μM (46–59 μg/L) compound spiked solutions: estrone (E1), 17β-estradiol (E2), 17α-ethynylestradiol (EE2), BPA; domestic wastewater.	E1 (0.44 μg/cm^2^), E2 (0.82 μg/cm^2^), EE2 (1.23 μg/cm^2^), BPA (0.32 μg/cm^2^). Higher concentrations causing membrane fouling.
MF [249]	zeolite imidazolate metal-organic framework (ZIF-8) nanoparticles incorporated into poly(tetrafluoroethylene) (PTFE) double layer polymer membrane.	hormones: progesterone (PGS) (0.5–5.0 mg/L); waste streams	95% PGS. High adsorption capacity and fouling tolerance, high porosity, low cost, efficient regeneration, ease operation.
MF [179,250,251,252]	hybrid composite membranes: TiO_2_/PES, TiO_2_/PVDF.	diclofenac (25 mg/L), ibuprofen (100 mg/L); wastewaters.	Diclofenac: 68% in 120 min for TiO_2_/PES membrane; 55% in 120 min for TiO_2_/PVDF membrane. Ibuprofen: 65% in 120 min for TiO_2_/PES membrane; 45% in 120 min for TiO_2_/PVDF membrane. Recycle the photocatalyst TiO_2_.
MF-RO [252,253,254,255,256,257]	Hybrid hollow fiber MF-RO membranes: MF polysulfone, RO polyamide.	Pharmaceuticals: carbamazepine, diclofenac, atenolol, azithromycin erythromycin etc., and pesticides between 162–240 ng/L. wastewater treatment plant.	Pharmaceuticals and pesticides: 98% and 100% (MF permeate: higher than 100 ng/L, RO ng/L or below the LOQs). MF-RO 97% for the most pharmaceuticals. RO pesticides: 67% 90%, 88% for diazinon, diuron, and 2,4 D.78 and 99% for MCPA and other pesticides, 97, 98% for MCPA and mecoprop.
MF [258]	CNT composite PVDF membranes.	Triclosan (TCS), acetaminophen (AAP), ibuprofen (IBU) 1 mg/L.	10–95%, increase with number of aromatic rings (AAP/IBU/TCS).
NF and RO [24]	polyamide thin-film composite for both NF and RO.	analgesics and anti-inflammatory drugs (ketoprofen < MQL–314 ng/L, diclofenac 60.2–219.4 ng/L, propyphenazone 51.5–295.8 ng/L), b-blockers, antiepileptic drug carbamazepine 8.7–166.5 ng/L, antibiotics, lipid regulator (gemfibrozil), diuretic as hydrochlorothiazide (58.6–2548 ng/L). full-scale drinking water treatment plant (DWTP) using groundwater.	NF and RO membranes: acetaminophen (44.8–73%), gemfibrozil (50–70%) mefenamic acid (30–50%). carbamazepine, hydrochlorothiazide, propyphenazone and glibenclamide (>85%), ketoprofen, diclofenac, and sulfamethoxazole (R > 95%), sotalol and metoprolol as blockers (R > 90%).
UF with coagulation and disk filtration [259]	hollow-fiber PVDF UF membrane and spiral-wound polyamide type TFC RO membranes combined with coagulation and disk filtration (CC–DF).	Micropolluants: atenolol (ATE), carbamazepine (CBZ), caffeine (CAF), diclofenac (DIC), dilatin (DIL), florfenicol (FLO), and sulfamethoxazole (SMX), A pilot-scale municipal wastewater system.	UF membrane (<17%), the RO membrane high removal efficiencies (91–98%), especially for negatively charged micropolluants (i.e., DIC and SMX) compared to the noncharged micropollutatns (CBZ, CAF, DIL) and/or positively charged micropollutants.
UF/NF [260]	micellar-enhanced ultrafiltration (MEUF) with polyethersulfone (UF) and cellulose acetate, polysulfone–polyamide thin film (NF).	11 ECs: acetaminophen (ACET), metoprolol (MET), caffeine (CAF), antipyrine (ANT), sulfamethoxazole (SUL), flumequine (FLUM), ketorolac (KET), atrazine (ATR), isoproturon (ISOP), 2-hydroxybiphenyl (HYD) and diclofenac (DIC), 0.5 mg/L. Cork processing wastewater.	Cationic surfactants cetyl pyridinium chloride (CPC)/cetyl trimethyl ammonium bromide (CTAB), pH 7.9 for: ATR 62/65.8% and ISOP 68.8/67.5%, Retention 95/85%: DIC > KET > SUL > FLUM (accordingly to the pKa values).
UF [261]	thin-film composite, cross-linked aromatic polyamide top layer, and PT polyethersulfone membrane.	amoxicillin, naproxen, metoprolol and phenacetin.	The retention coefficients with the UF membranes followed the sequence naproxen > metoprolol > amoxicillin > phenacetin, and with the NF membranes:amoxicillin > naproxen > metoprolol > phenacetin.
NF [262,263]	commercial NF-270, 800 kPa pressure.	Carbamazepine, BPA, triclosan, butyl benzyl phthalate, and 4- nonylphenol (100 ng/L). Untreated wastewater from agricultural and urban wastes. Hormones and tert-butyl phenol secondary wastewater.	Removal increased for hydrophobic compounds due to adsorption onto membranes (>90%), while water solubility reduced the retention of BPA. Hormones and tert-butyl phenol removal up to 90%.
NF [264]	commercial NF-90 and NF-270 membranes.	sulfamethoxazole, diclofenac sodium, hydrochlorothiazide, 4-acetamidoantipyrine, nicotine and ranitidine hydrochloride. Wastewater streams.	Solute retention for NF-90: >95%, NF-270: from 75% (for nicotine) to 95% (for ranitidine hydrochloride).
NF [265]	polyamide membrane (comparison with polysulfone, polyester membranes).	estrone and estradiol Aqueous solutions.	Polyamide NF membranes the highest hormone adsorption.
NF [266,267]	grafted polyamide membranes with methacrylic acid cross-linked with ethylene diamine (ED).	Pharmaceutically active compounds: BPA, ibuprofen and salicylic acid.	95% rejection for BPA, 74% rejection with pristine membrane.
NF [268]	NF hollow fiber membrane dry-jet wet spinning using a hyperbranched polyethyleneimine (PEI) as cross-linker.	20 ppm CPF. Synthetic solution.	pH 3 and positively charged PEI modified NF hollow fiber membranes: 99% rejections. pH increased with rejection decreased (CPF molecules become less positively charged).
NF [269]	thin polyamide skin layer on top of a microporous polysulfone support.	sulfamethoxazole, carbamazepine, and ibuprofen. Pharmaceuticals spiked, 500 g/L.	Sulfamethoxazole and ibuprofen (negatively charged) retention increased with ionic strength increasing.
NF [270]	commercial NF membrane based on TFC.	norfloxacin (NOR), ofloxacin (OFL), roxithromycin (ROX), azithromycin. Wastewater treatment plant.	98% rejections. UV/O_3_ process, removal efficiencies: 87%, with 40% dissolved organic carbon (DOC), 58% acute toxicity reduction.
